# Remote Patient Monitoring for Global Emergencies: Case Study in Patients With COVID-19

**DOI:** 10.2196/66773

**Published:** 2025-07-18

**Authors:** Ramin Ramezani, Wenhao Zhang, Minh Cao, Alex Bui, Antonia Petruse, Amelia Weldon, Arash Naeim

**Affiliations:** 1 Department of Computer Science, University of California, Los Angeles, 550, 404 Westwood Plaza, Engineering 6, Los Angeles, CA, 90095, United States, 1 4242997051; 2 Department of Bioengineering, University of California, Los Angeles, Los Angeles, CA, United States; 3 Clinical and Translational Science Institute (CTSI), University of California, Los Angeles, Los Angeles, CA, United States

**Keywords:** COVID-19, physical activity, health care delivery models, wearable sensors, indoor localization, Bluetooth Low Energy beacons, smartwatches, mobile phone, remote sensing technology

## Abstract

**Background:**

The COVID-19 pandemic has highlighted the critical need for telehealth and remote patient monitoring in health care delivery. Despite the growing use of on-body wearable sensors for continuous monitoring and predicting adverse events, their widespread adoption remains a significant challenge. While the pandemic has accelerated the acceptance of these technologies, achieving widespread integration requires their sustained incorporation into routine health care practices beyond emergencies. In this study, we extend the application of our previously developed remote patient monitoring system to patients with COVID-19.

**Objective:**

Our objective is to assess whether the metrics obtained from our previously developed system can provide additional insights into the recovery trajectory of individuals affected by COVID-19. This case study aims to demonstrate that remote patient monitoring systems can be adapted to diverse patient cohorts during emergencies. We aim to illustrate the ease of deployment, particularly when these systems are already integrated into the existing health care ecosystem.

**Methods:**

From November 2020 to July 2021, a total of 73 patients were recruited through the University of California, Los Angeles, Center for Smart Health, after having consented to participate in this study for 2 weeks. The research concentrated on an exploratory analysis, focusing on the detailed examination of characteristics and behaviors of patients with COVID-19 as captured by the remote patient monitoring system. We collected day-to-day changes in the following sensor measurements: daily activity, daily energy expenditure, indoor localization, SpO_2_, respiratory rate, heart rate, and temperature.

**Results:**

Out of the 73 patients satisfying the inclusion criteria, 41 successfully adhered to using the monitoring technology, with only 22 providing substantial watch data (>4 h). Among the participants, 39 used the pulse oximeter, 37 used the thermometer, and 36 used respiratory monitoring at night. This study demonstrated an overall increase in patients’ activity levels toward the end of this study, with many beginning to leave their homes after 2 weeks. Additionally, respiratory rates shifted toward healthier lower levels, and oxygen saturation improved. Fatigue and headache were identified as the most prevalent symptoms, followed by cough and loss of smell.

**Conclusions:**

The conclusion highlights the critical importance of monitoring patients outside of hospital settings, especially during pandemics, when patients travel to hospitals or receive home visits by health care professionals, which could increase the risk of disease transmission. Studies demonstrating the benefits and efficacy of remote monitoring in home settings can better prepare health care professionals for future pandemic events. Continuous monitoring of a wide range of patient metrics, from activities to vital signs, and integration of these data into electronic health records would not only improve accuracy and reduce the burden of data collection but also pave the way for enhanced home care, offering higher quality care at a lower cost.

## Introduction

In late 2019, an outbreak of COVID-19 emerged and spread rapidly worldwide. As of May 5, 2020, when we began this study, there were more than 3.6 million confirmed cases and >250,000 deaths attributed to COVID-19 worldwide, with over 69,000 deaths alone in the United States. These numbers have reached 7 million deaths worldwide, including 1.2 million in the United States alone [1,2]. Despite the development of multiple vaccines, the virus and its new variants continue to wreak havoc in the public health sector. While the mortality rate for COVID-19 was reported to be around 2%, the number was significantly increased among older patients with underlying coexisting conditions. Notably, in hospitalized patients, the death rate approached 15%. Individuals who were at mild to moderate risk, such as those with cancer, could demonstrate significant clinical deterioration within 24‐48 hours.

In recent years, during the COVID-19 pandemic, we observed the implementation of policies such as “shelter in place” aimed at preventing the unnecessary strain on the health care system, measures that may become necessary in the face of another pandemic. Some high-risk patients were instructed to “weather the storm” at home without adequate monitoring, despite the potential for rapid deterioration in their health [3]. Over the past decade, the emergence of commercially available, affordable, and lightweight sensors has significantly accelerated the adoption of remote patient monitoring systems within the health care system for continuous and comprehensive patient tracking. Even before the COVID-19 pandemic, substantial evidence indicated that continuous monitoring of vital signs, such as pulse oximetry and heart rate, was associated with reduced mortality [4,5]. This aligns with numerous studies conducted during the pandemic, which explored the use of pulse oximetry and other wearable devices for continuous vital sign monitoring [4,6,7]. During the COVID-19 pandemic, numerous studies evaluated the use of technology for patient management and assessed its effectiveness in reducing hospitalizations [3,6,8-11]. While some initiatives were labeled as remote patient monitoring, they often focused more on telehealth, e-visits, and the use of patients’ existing technologies, such as smartphones to connect to health care professionals. These studies demonstrated encouraging results, indicating that technology can significantly enhance patient management [6,8,9].

In a series of studies, we had introduced and documented the development and implementation of our remote patient monitoring system and its clinical validation within older patient populations at risk of various health conditions [12-15]. This platform, known as Sensing At-Risk Population (SARP), encompasses activity monitoring through smartwatches, indoor localization using stationary beacons, and the collection of additional physiological data via wireless sensors. Data are securely and automatically transmitted to a Health Insurance Portability and Accountability Act (HIPAA)–compliant cloud infrastructure. For this study, we expanded the capabilities of our system to serve as a monitoring hub and to support additional devices tailored to the needs of COVID-19 positive patients. We incorporated commercially available Bluetooth-enabled thermometers, pulse oximeters, and respiratory distress monitors. These additions enabled us to capture relevant information for monitoring and assessing COVID-19 positive cases. The primary objective of this study was to enhance existing outpatient COVID-19 positive clinical trials, which incorporate the variables that our system captures as secondary or exploratory endpoints. By leveraging our platform, we aimed to minimize COVID-19 positive patients’ exposure while collecting crucial vital information. We intended to gather this data within a natural environment, shedding light on the diverse array of symptoms presented by COVID-19 positive patients, thus contributing to a better understanding of this disease and the ongoing pandemic using the emerging remote patient monitoring systems.

## Methods

### Design, Setting, and Participants

Of study design and participants, patient recruitment occurred from November 2020 to March 2021 at the University of California, Los Angeles (UCLA) Health Center. We emphasized that participation in the study would not affect their care at UCLA and was entirely separate from their medical treatment. Participation would conclude either after 2 weeks or if the patient was admitted to the hospital during that timeframe. This study’s protocol was obtained from the Institutional Review Board of UCLA.

Eligible participants were individuals aged 18 years or older with a COVID-19 diagnosis who were not hospitalized or had known exposure to a COVID-19 positive case. They required the ability to manage their condition at home, access to home Wi-Fi, proficiency in either English or Spanish, and a willingness to provide informed consent by signing the approved form (IRB #20‐001565) from the UCLA, titled “Early Detection of Health Improvement and Decline Through Remote Health Monitoring in COVID-19 Positive Patients.”

### Inclusion and Exclusion Criteria

Inclusion criteria were wearable device compatibility (ability to wear a watch), willingness to host the remote monitoring system for 2 weeks, aged 18 years or older, current UCLA Health patient, confirmed positive COVID-19 laboratory result, or known exposure to COVID-19.

Exclusion criteria were clinical diagnosis of movement disorders (eg, Parkinson Disease) and failure to meet inclusion criteria.

### About SARP

SARP is a remote patient monitoring system developed by UCLA’s Center for SMART Health, designed to cater to patients beyond the confines of health care institutions. Its primary purpose is to monitor vulnerable, at-risk populations by simulating the measurement of activities of daily living and instrumental activities of daily living [16] through cost-effective sensor technology. SARP has been used to generate prognostic data and predictive models for mortality and functional decline [12-15].

The core components of SARP encompass hardware, including an Android Smartwatch and readily available proximity Bluetooth Low Energy (BLE) beacons, and clinically validated software featuring activity recognition and indoor localization algorithms. Additionally, SARP incorporates a remotely triggered adaptive smart questionnaire mechanism, data visualization tools, and algorithms for assessing frailty, all within a HIPAA-compliant infrastructure [17].

Activity features were derived from three groups of parameters using smartwatches and BLE beacons: (1) activity recognition (eg, sitting time or standing time), (2) indoor localization (eg, time in bed or time in the bathroom), and (3) raw acceleration quantification (mean absolute deviation [MAD] in accelerometer signal). By combining these attributes, we created features such as sitting time in bed and energy expenditure during walking or while in bed [12,13].

To ensure fair comparisons among patients with different watch wearing times, we normalized features by dividing the time spent on activities or in locations by the total wear time (uptime). Energy-related features were also normalized by uptime to yield energy intensity and by the total daily value to calculate energy percentage.

BLE beacons are used in the SARP system to estimate indoor patient locations by measuring the received signal strength indicator values via smartwatches [18-20]. Patients were instructed to place a BLE beacon in each designated indoor location: the kitchen, bathroom, bedroom, dining room, and television or sitting room. If the system does not detect any beacons, it infers that the patient is outside, indicating they are not at home.

### Ethical Considerations

This study was approved by the UCLA Ethics Review Board (IRB #20‐001565), ensuring adherence to ethical standards and the protection of human participants’ rights and welfare. Participants were recruited through oral consent, which included a clear explanation of the study’s purpose, duration, procedures, potential risks (such as discomfort from wearing a smartwatch or placing sensors at home), and confidentiality measures. Patients were also informed that the data collected could be used for future research studies, may be shared with other investigators, or used in collaboration with the private sector to develop smart algorithms, without requiring additional consent. Data collected were anonymized and deidentified before analysis, following strict confidentiality protocols. No financial compensation was offered to participants. Additionally, no identifiable images or data of participants are presented in this paper or supplementary materials.

### Data Collection

For this study, SARP was modified to integrate a series of US Food and Drug Administration (FDA)–cleared, Bluetooth-enabled devices, including pulse oximeters, thermometers, and ultra-wideband radar technology for respiratory monitoring. The utility of these metrics for patient assessment has been demonstrated in numerous studies, with further research validating the accuracy of Bluetooth-enabled devices for home and remote monitoring [5,7,9,10,21-24]. While evaluating the efficacy of these devices is beyond the scope of this paper, we assume their reliability, given that pulse oximeters, thermometers, and respiratory monitoring devices incorporated into the SARP system are FDA-cleared. However, it is worth noting that some studies indicate ultra-wideband radar may overestimate respiratory rates at low levels and underestimate them at high levels [25,26]. While FDA clearance validates accuracy in controlled settings, real-world performance may vary due to user compliance, environmental factors, and device positioning. Our study did not independently validate these devices but relied on their FDA clearance as a baseline for integration into remote monitoring workflows.

Among the integrated devices, the smartwatch was designed for continuous monitoring, inferring metrics such as activity tracking, energy expenditure, and time spent in various indoor locations. In contrast, other devices were used on a spot-check basis, voluntarily recorded by patients. Patients were advised to use the pulse oximeter and thermometer at least twice daily, while the respiratory monitoring device was mounted next to the bed for continuous tracking whenever the patient was present.

All data were securely uploaded to a HIPAA-compliant Azure internet of things cloud infrastructure, expanding the SARP system’s remote patient monitoring capabilities. The integration of Bluetooth-enabled devices allowed for the remote collection of additional vital data, complementing existing infrastructure. Specifically, for this project, pulse oximeters and thermometers were used to detect potential COVID-19 symptoms, such as shortness of breath and fever, aiding in testing recommendations. Additionally, SARP incorporated an FDA-cleared Bluetooth device equipped with radar, a microphone, and a light sensor to monitor respiratory function, enabling the detection of hypoventilation, hyperventilation, and impaired lung function. This device was recommended to be placed adjacent to the patient’s bed, primarily for nighttime monitoring or when the patient was in the room.

The devices used included the AndesFit Non-Contact Forehead and Surface Thermometer with Bluetooth 4.0 (ADF-B38A), AndesFit Pulse Oximeter with Bluetooth 4.0, Circadia ultra-wideband Respiration Monitoring Device, TicWatch Pro 3 Smartwatch with Bluetooth module, Proximity Beacons (MCU ARM Cortex-M4 32-bit processor with floating-point unit), and Amazon Fire Tablet 7.

We recorded the day-to-day changes in the following sensor measurements and compiled the assessments shown in Textbox 1:

1. Position: lying down, sitting, standing, and walking inferred from continuous accelerometer data collection.
2. Active: active (walking), active (not walking), and nonactive inferred from continuous accelerometer data collection.
3. Steps: total daily steps is the total distance traveled inferred from continuous accelerometer data collection.
4. Indoor localization: bedroom, bathroom, kitchen, dining room, family room, and office inferred from accelerometer data and indoor location beacon received signal strength indicator scans during and immediately after each movement detected by the smartwatch.
5. Sleep quality: duration and toss and turn, inferred from continuous accelerometer.
6. Heart rate: spot check by patient using pulse oximeter.
7. Temperature with infrared technology spot check by the patient using a Bluetooth thermometer.
8. SpO_2_ oxygen saturation spot check by the patient using pulse oximetry.
9. Breaths per minute measured using ultra-wideband radar technology, captured during sleep by a device positioned next to the patient’s bed.
10. Respiration waveform (inhalation or exhalation patterns) with ultra-wideband radar technology.

Textbox 1. **Patient assessments from sensor measurements.**

Summary sensor assessments

- Percent of time out of bedroom
- Percent of time out of house
- Percent of time walking
- Total daily steps
- Walking speed
- Counts of activities and locations
- Total active time
- Total energy
- Weighted total activity score
- Spot check of temperature
- Spot check of SpO_2_
- Spot check of average breaths per minute
- Respiratory rate and chest displacement
- Early warning score

As described in the study by Ramezani et al [12], active or nonactive is determined by an empirical threshold of 0.02 m/s^2^ (2 cm/s^2^) imposed on the MAD value of an accelerometer signal every 10 seconds. The threshold translates to 1 meter of displacement of the hand in 10 seconds (hand wearing the smartwatch with accelerometer). Furthermore, the energy expenditure is estimated to be proportional to the MAD of the accelerometer magnitude signal.

### Exploratory Analysis

The primary objective of this study was to conduct an observational analysis aimed at developing predictive models for forecasting adverse COVID-19 outcomes, including hospitalization within 24 or 48 hours. Given the absence of adverse events among the participants, the research emphasis shifted toward an exploratory analysis, concentrating on the delineation of characteristics and behaviors of patients with COVID-19 as captured by the remote patient monitoring system.

All the analyses were performed using the Python Programming Language (version 3.11.3; Python Software Foundation) libraries, Pandas (version 2.2.1), NumPy (version 1.25.2), SciPy (version 1.11.1), Scikit-learn (version 1.3), and Seaborn library (version 0.12.2) were used for statistical data visualization.

### Analytics Inclusion Criteria

To ensure the meaningfulness of the activity data, we imposed an additional inclusion criterion requiring a minimum daily wear time of the smartwatch of at least 4 hours. However, the use of the remote patient monitoring system kit was not limited solely to the smartwatch; it also included a thermometer and pulse oximeter for spot checks, along with a respiratory monitor capable of continuous data capture in the bedroom. Data collection was conducted either continuously or at specific intervals from these devices. For analysis, we included individuals with measurements taken on more than 7 distinct days and calculated the mean value of the metric for each day.

### Activity and Energy

Energy intensity, as detailed in the SARP section, was analyzed for all individuals on their first day of the study (baseline) and their last day using a box plot. The plot displays the median line, representing the central tendency, and the overall distribution of energy intensity values. It is important to note that these observations were made after applying the inclusion criteria, which only considered days where patients had more than 4 hours of watch wearing time.

Using Bluetooth beacons, the time each patient spent in specified locations within their home was calculated to better form a daily storyline of their activity patterns. The goal was to assess whether patients spent less time in the bedroom as this study progressed and whether they spent more time outside their residence. The percentage of time spent in each location was calculated by dividing the duration in that location by the total uptime (watch wearing time) for that day. A heatmap was generated to visualize the average percentage of time spent in each location longitudinally over 2 weeks.

It is important to note that data were analyzed after applying inclusion criteria, and some participants were in this study for fewer than 2 weeks. For each day of this study, the time spent in each location was averaged across the number of patients observed on that particular day. Given that the start and end dates varied across patients, the analysis was conducted by calculating each patient’s individual day. For example, “day 1” represents the average time percentage spent in each location for all patients on their respective first day, which may differ chronologically between patients.

### Vital Signs

The distributions of SpO_2_, pulse rate, respiratory rate, and temperature were visualized using kernel density estimate (KDE) plots. The KDE plots depict patients’ baseline (the first day of data collection) and their final day (the last available day of this study). For each patient, the mean value of observations was used for both the baseline and the final day. A minimum interval of 7 days between the baseline and final day was enforced to exclude patients with only a few days of data, as their differences could not accurately represent longitudinal trends. It is important to note that the KDE visualization may include out-of-bounds values due to the edge effect, where Gaussian distribution-based estimation extends beyond the actual data range. When the observations are close to the edges of the data range, the KDE, which is formed by centering around each data point, may extend beyond the boundary. This can be addressed by truncating the graph and clipping the x-axis out-of-bound values, although this is merely a visualization adjustment. Alternatively, the edge effect can be mitigated by smoothing the KDE curve through bandwidth adjustment. In the Seaborn library, the default bandwidth value is determined by Scott rule (set to 1) [27] and automatically adapts to the data characteristics [28]. While increasing the bandwidth reduces sensitivity to individual observations and produces a smoother curve, the authors opted not to adjust the bandwidth due to the limited number of observations and the need to maintain the graph’s sensitivity to changes.

### Self-Reported Survey

Daily self-reported surveys, collected using the SARP app provided to patients on tablets, were integrated with sensor data. The purpose of this integration was to later align the data with potential complications, including cardiovascular issues or hospitalizations, to retrospectively investigate whether any early indications could have been inferred from the observational data. This analysis aimed to identify potential early warning signs and develop timely interventions.

## Results

### Demographic Characteristics

Of 176 interested patients who were approached by our team, 73 met the eligibility criteria and were enrolled to use our remote patient monitoring kit, SARP, as detailed in Table 1. Of these, 41 patients successfully connected to and complied with the system.

**Table 1.** Sociodemographic characteristics of the patient cohort (N=73).

Characteristics	Patients, n (%)
Race	
Asian	3 (4.1)
Black or African American	6 (8.2)
Hawaiian or Other Pacific Islander	1 (1.4)
White	42 (57.5)
Other	21 (28.8)
Ethnicity	
Not Hispanic or Latino	44 (60.3)
Hispanic or Latino	26 (35.6)
Unknown or not reported	3 (4.1)
Sex	
Female	38 (52)
Male	31 (42.5)
Prefer not to say	4 (5.5)
Highest education level	
High school diploma	6 (8.2)
Some college	19 (26)
Bachelor’s degree	28 (38.3)
Graduate degree	18 (24.7)
No response	2 (2.73)
Employment status	
Employed	44 (69.8)
Unemployed	10 (13.7)
Self-employed	8 (11)
Retired	4 (5.5)
Marital status	
Married	34 (46.6)
Divorced	2 (2.7)
Widowed	2 (2.7)
Single	29 (45.3)
Separated	2 (2.7)
Age (years)	
20‐30	14 (19.2)
30‐40	15 (20.5)
40‐50	17 (23.3)
50‐60	14 (19.2)
60‐70	9 (12.3)
70‐80	3 (4.1)
80‐90	1 (1.4)
Weight (lbs)	
110‐150	20 (27.3)
150‐190	17 (23.3)
190‐230	24 (32.9)
230‐270	9 (12.3)
270‐310	2 (2.8)
310‐360	1 (1.4)
Not reported	1 (1.4)
Height (feet)	
3‐4	1 (1.4)
4‐5	15 (20.5)
5‐6	56 (76.7)
Not reported	1 (1.4)

To reiterate, the SARP remote patient monitoring system included a smartwatch, thermometer, pulse oximeter, and a respiratory rate monitoring device. We established an additional inclusion criterion that mandated a minimum daily wear time for the smartwatch of at least 4 hours.

There were 22 patients with substantial watch data (>4 h per day) with an average usage of 10.1 (SD 4.3) days of watch wearing time. A total of 39 patients used a pulse oximeter to check the SpO_2_ and heart rate, with an average usage of 10.1 (SD 4.7) days of using the device. The number of patients who used the thermometer decreased to 37 with an average usage period of 9.7 (SD 4.8) days. The number of patients with respiratory data is 36, with an average of 11.4 (SD 4.7) nights of continuous monitoring of breathing rate per minute.

### Activity and Energy

**Figure 1.**
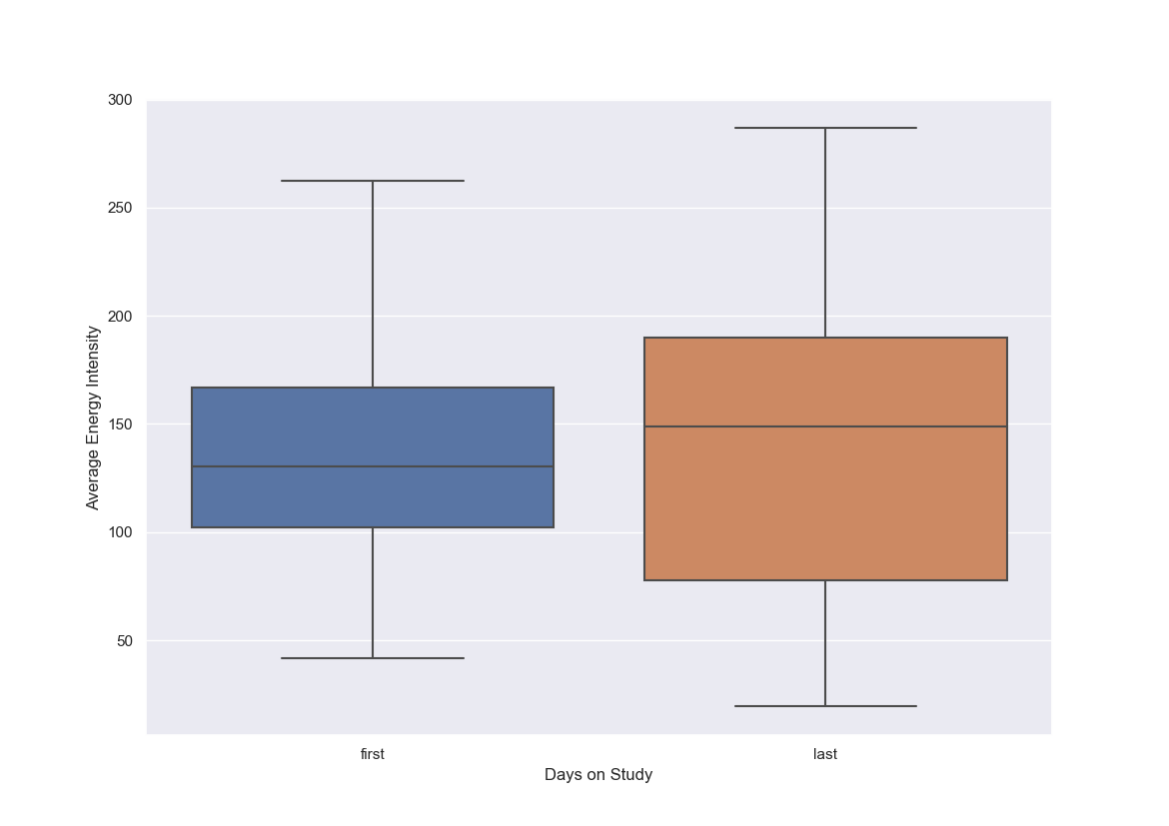
Average energy intensity: first day versus last day.

The box plot in Figure 1 shows that the overall energy levels of patients increased on the last day of this study compared to the first day. Both the mean intensity and the variability of energy levels showed an upward trend. The figure indicates an overall improvement in the physical activity of patients with COVID-19 over time, though with varying degrees of recovery across the patients.

**Figure 2.**
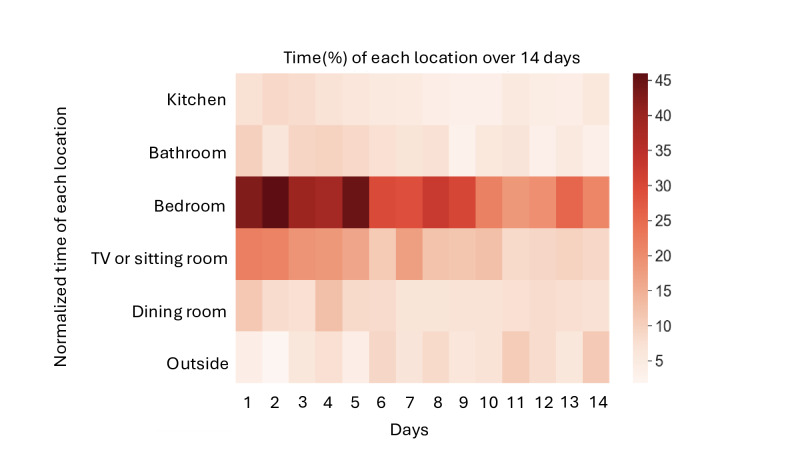
Heatmap of patients' activities during the 2-week study.

By analyzing the heatmap of patient activities shown in Figure 2, it is evident that toward the end of this study, patients were expending less time in the bedroom and more in other areas outside the home. The fading color in the bedroom area toward the end of this study suggests a decrease in time spent in that location. Similarly, there is a noticeable decline in activity within other home settings, such as the television or sitting area, as captured by indoor localization beacons. This trend likely reflects patients’ gradual recovery and increased mobility, allowing them to engage more in activities outside their homes.

### Vital Signs

**Figure 3.**
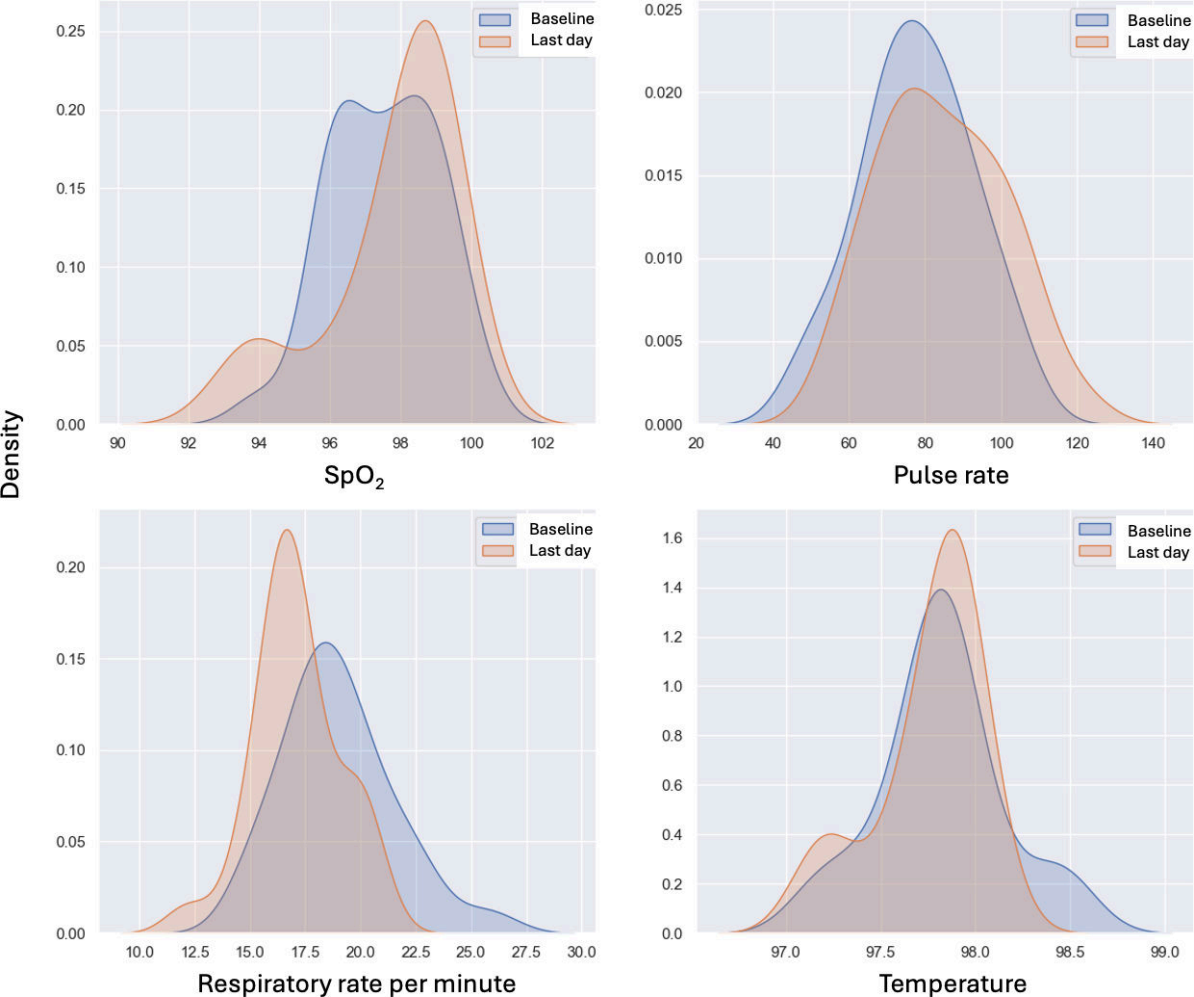
Biomarkers: first day versus last day.

In Figure 3, KDE graphs for 4 vital signs show that the distribution for SpO_2_ has shifted slightly to the right from baseline to the last day, indicating an overall improvement in oxygen saturation levels by the end of this study. The value of the last day’s SpO_2_ distribution is higher than the baseline, suggesting more patients reached higher SpO_2_ levels on the last day.

The pulse rate distribution on the last day shows a slight shift to the right compared to the baseline. This suggests that patients’ pulse rates tended to increase slightly by the end of this study. The distribution also appears to broaden slightly, indicating greater variability in pulse rates among patients on the last day.

On the respiratory rate subgraph, there is a noticeable shift in distribution toward lower rates on the last day compared to the baseline. This may indicate that, on average, patients had a lower respiratory rate by the end of this study, potentially reflecting improved respiratory function. The last day’s distribution is narrower, suggesting reduced variability in respiratory rates, with most patients converging around healthier respiratory measures.

The temperature distributions, however, remain relatively similar in both baseline and the last day, with only slight differences.

### Self-Reported Survey

The tablet provided to each patient, serving as a device hub, was also available for completing protocol-mandated surveys. Participants could voluntarily report their symptoms and overall wellness at least once daily. The survey responses are included in Multimedia Appendix 1, which illustrates the prevalence of symptoms reported by patients at least once during the 2-week observation period.

Multimedia Appendix 1 indicates that fatigue (22/75, 30%) and headache (22/75, 30%) were the most prevalent symptoms among patients with COVID-19, followed by cough (19/75, 25%) and loss of smell or taste (20/75, 26%). Despite these symptoms, only 6% (5/75) of patients contacted a health care provider, suggesting either mild severity or potential difficulties in seeking medical advice. As there was a direct contact number for the patients in this study and the ease of contacting them, it would be safe to assume that mild severity was the main reason. This underscores the need for careful monitoring of common COVID-19 symptoms. Other symptoms were chest pain (12/75, 16%), shortness of breath (10/75, 13%), fever (6/75, 8%), runny nose (17/75, 22%), diarrhea (10/75, 13%), and body aches (15/75, 20%).

## Discussion

### Principal Findings

Our analysis of activity and indoor location suggests that a positive trend in data from wearable and remote monitoring devices is aligned with the recovery of patients with COVID-19 over this study’s period. It is important to emphasize that the lower numbers in each category (smartwatch, pulse oximeter, thermometer, and respiratory monitoring) compared to the 73 participants result from not meeting the analytics inclusion criteria. This includes insufficient compliance with smartwatch wear time, voluntary use of Bluetooth-enabled devices for spot checking, and willingness to use the respiratory monitoring device next to their bed.

Although we did not directly ask patients about their compliance or the reasons for not using the system more frequently in this study, insights from our previous studies using the same infrastructure [12]—albeit with a different cohort (at-risk older population)—suggest that the bulkiness of smartwatches was a primary issue. Most commercially available smartwatches, despite recent advancements in sensor quality approaching medical-grade standards, remain relatively bulky due to their large battery packs. This design choice enables them to perform various functions, including connectivity with phones, but makes them less suitable for medical use, where comfort and continuous wearability are crucial. In short, most smartwatches are not specifically designed for patient use, and the current battery technology often results in devices that are bulky and somewhat inconvenient for continuous wear. In contrast, other sensors, such as temperature monitors and pulse oximeters, were used more frequently, as they were used for spot checks rather than requiring continuous wear, making them more acceptable to patients.

Figure 1 shows an increase in overall energy levels, with both the mean energy intensity and variability rising from the first to the last day, indicating an overall improvement in physical activity. Figure 2 offers additional insights, revealing that patients progressively expended less energy in the bedroom and other indoor locations, such as the television or sitting area, as this study progressed. This decline in indoor activity suggests that patients were recovering and becoming more mobile, with greater energy expenditure in locations outside their homes. The shift in activity distribution likely reflects improved physical health, as indicated by the higher intensity color in the heatmap corresponding to outdoor locations toward the end of this study.

Our study results are consistent with a systemic scoping review of studies published between 2019 and 2022, in which smartwatches or fitness trackers were reported as the preferred type of wearable technologies for early and presymptomatic detection [29-32]. Much of the published literature is focused on using wearables for early detection [33,34]. These studies do show that wearables can track heart rate and heart rate variability with improvement over 7 days [35]. Moreover, SpO_2_ and activity tracking from wearable devices might be able to help identify patients with COVID-19 at risk for sudden death [36] based on a systematic review. However, the best results are looking at physiological features in a multimodal approach where one can achieve sensitivity as high as 90% and specificity as high as 80% [37]. Our study asked about self-reported symptoms and the importance of combining symptoms with sensor data that has been shown to allow for better predictive models [38]. The use of wearables and remote monitoring has also been explored in managed long COVID-19 [39].

The prevalence of fever among patients with COVID-19 varies across studies and cohorts, with a high percentage observed in hospitalized patients [40]. However, in nonhospitalized patients, fever is less frequently observed or may be entirely absent. In studies such as the one by Shi et al [41], fever was recorded for fewer than 2 days throughout the illness, primarily in frail patients, even after lowering the temperature threshold from 100.4°F to 100°F. Body temperature in our cohort never exceeded 98.6°F. Figure 3 indicates that patients’ body temperatures were relatively stable throughout this study, with a marginal increase by the last day. Both distributions of body temperatures in baselines and the last day are tightly clustered, indicating consistent body temperatures across patients. This suggests that either the cohort did not exhibit fever, or the noncontact infrared thermometers used were not sufficiently sensitive, despite being FDA-cleared. This aligns with an FDA study [42] highlighting misleading labeling of FDA-cleared devices during COVID-19, showing that noncontact infrared thermometers may fail to reliably detect fevers when used on adults and may not meet the accuracy specifications advertised in manufacturers’ instructions for use. Notably, 8% of patients in this study reported fever in the self-reported surveys (Multimedia Appendix 1), indicating that the subjective experience of fever was prevalent in at least 8% of the cohort.

Figure 3 of vital signs overall suggests that by the end of this study, patients generally showed signs of physiological improvement, with higher SpO_2_ levels, slightly elevated pulse rates, and lower respiratory rates. Such trends indicate a recovery or improvement in the monitored patients’ vital signs.

### Study Limitations

Patient compliance with wearing a smartwatch and using the Bluetooth-enabled thermometer and pulse oximeters was one of the main challenges of this study, and we anticipate this to be a common obstacle in similar studies that aim to use wearable technology for patient monitoring. In contrast, the ultra-wideband respiration monitoring device required no patient interaction and could monitor respiration passively. However, the accuracy of this device could be compromised if more than 1 person were in the bedroom or if the patient left the room. To mitigate this, we ensured that only the results from patients who lived alone in the monitored bedroom were included in the analysis. While setting up the devices was designed to be straightforward—requiring only a tablet and a simple app we provided to connect all devices to the patients’ home internet in 1 step—it is reasonable to assume that certain patients, particularly those less familiar with technology, may have found the setup process challenging.

As the cohort was randomly selected, the predominance of younger participants indicated in Table 1 may reflect self-selection bias, accessibility to technology, or willingness to participate in remote monitoring studies rather than a deliberate sampling choice. However, this demographic skew could impact the relevance and generalizability of the findings, particularly if younger individuals have different health profiles, technology engagement levels, or adherence behaviors compared to older populations. To address this limitation, we acknowledge that further studies with more diverse age representation are needed to validate the findings across broader demographics, particularly in populations more likely to benefit from remote health monitoring, such as older adults or individuals with chronic conditions.

### Conclusions

The COVID-19 pandemic demonstrated the importance of telehealth and remote monitoring of at-risk patients. A recent cost-utility analysis estimates that daily pulse oximetry use with a follow-up after 3 weeks could reduce the mortality rate to 6 per 1000 patients, compared to 26 per 1000 without at-home monitoring. Various studies suggest that remote patient monitoring in patients with COVID-19 could potentially reduce hospitalizations and deaths by as much as 80% and yield cost savings of around $12,000 per patient [10,43]. This underscores the growing importance of using technology for continuous at-home monitoring, which can enhance the quality of care while significantly reducing costs.

In this study, we demonstrated that affordable remote monitoring devices can effectively track individuals over time, revealing trends in their health status. However, a significant challenge in remote and home monitoring systems is ensuring participant adherence. Our study found that only 41 of 73 individuals consistently used all the provided devices. This finding aligns with previous research, which suggests that patients are more likely to engage in remote monitoring or mHealth when they perceive an immediate personal benefit. Based on anecdotal feedback, patients appeared to prefer passive sensing over more demanding active monitoring. However, no formal assessment of patient satisfaction was conducted.

This study also generated novel data specific to COVID-19, comparing self-reported symptoms with objective monitoring data. Additionally, the findings highlight the potential benefits of implementing remote patient monitoring systems in future pandemics, as they provide continuous, scalable, and contactless health tracking, which could aid in early detection, triage, and disease management while minimizing the burden on health care facilities.

## Supplementary material

10.2196/66773 Multimedia Appendix 1 Prevalence of symptoms and health care contact among patients.

## Abbreviations

BLE: Bluetooth Low Energy
FDA: US Food and Drug Administration
HIPAA: Health Insurance Portability and Accountability Act
KDE: kernel density estimation
MAD: mean absolute deviation
SARP: Sensing At-Risk Populations
UCLA: University of California, Los Angeles
