# Supplementary figures and images for: Remote Patient Monitoring for Global Emergencies: Case Study in Patients With COVID-19
Source: JMIR Form Res. 2025 Jul 18;9:e66773. doi: 10.2196/66773 (PMC12294641; doi:10.2196/66773)

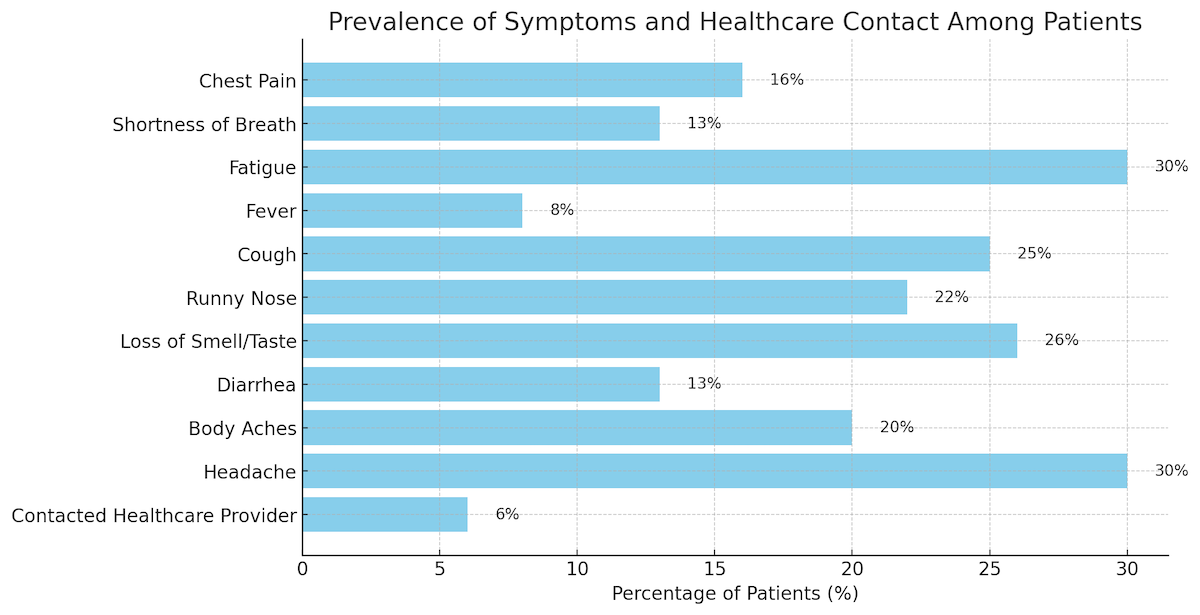

Supplement: Multimedia Appendix 1 [file formative-v9-e66773-s001.png]
